# Aging Behavior of EPDM Compounds with Ground Tire Rubber (GTR) as a Functional Substitute for Calcium Carbonate

**DOI:** 10.3390/polym18030367

**Published:** 2026-01-29

**Authors:** Philippe Rotgänger, Vanessa Spanheimer, Danka Katrakova-Krüger, Ulrich Giese

**Affiliations:** 1 Materials Laboratory, Faculty of Computer Science and Engineering Science, TH Köln, 51643 Gummersbach, Germanydanka.katrakova-krueger@th-koeln.de (D.K.-K.); 2 Deutsches Institut für Kautschuktechnologie e. V., 30519 Hannover, Germany; ulrich.giese@dikautschuk.de

**Keywords:** ground tire rubber (GTR), end-of-life tires (ELTs), rubber recycling, calcium carbonate replacement

## Abstract

This study investigates the substitution of calcium carbonate (CaCO_3_) with ground tire rubber (GTR) in EPDM-based elastomer formulations as a strategy for sustainable material development. Unlike conventional approaches, this work employs GTR as a direct filler replacement. Temperature scanning stress relaxation (TSSR) analyses confirm that GTR participates in vulcanization. Initial incorporation of GTR reduces crosslink density (CLD) and mechanical performance due to structural defects, while accelerators present in the recycled phase promote faster curing. This study focuses on the aging behavior of the compounds to evaluate possible long-term effects on the material. The thermo-oxidative stress leads to further crosslinking, resulting in higher CLD, increased stiffness and reduced elongation at break. Overall, partial replacement of CaCO_3_ by GTR proves feasible, offering a balanced compromise between sustainability and performance, whereas high GTR contents significantly impair mechanical properties.

## 1. Introduction

The recycling of crosslinked elastomers remains a major challenge in polymer materials science, as their thermoset-like structure prevents remelting and reshaping [1]. Among the available recycling strategies, mechanical grinding of end-of-life tires (ELTs) to produce ground tire rubber (GTR) offers a scalable and economically viable approach [2,3,4]. However, GTR incorporation into new rubber formulations remains limited due to its detrimental effects on mechanical properties, particularly when used as a polymer substitute or secondary filler [2,5,6,7].

Most studies treat GTR as an inert or partially devulcanized material that dilutes the polymer matrix, often in minor proportions to limit deterioration of tensile strength and elongation [2,6,7,8,9]. While several works explore devulcanization or compatibilization strategies to improve GTR–matrix interaction [8,9,10,11,12,13], the potential chemical reactivity of GTR during vulcanization remains largely unexplored [10]. In particular, the diffusion of curatives (e.g., sulfur) from the matrix into GTR particles can significantly influence the crosslink structure and kinetics but has rarely been quantified [5,6,14,15].

Sulfur migration can result in local inhomogeneities in crosslink density (CLD), especially since sulfur present in the matrix is free and mobile, while the sulfur in GTR is primarily bound within its original network. This mobility imbalance can lead to a net diffusion of sulfur from the matrix into the GTR phase, lowering the available curatives in the continuous phase and thus reducing overall CLD and mechanical integrity [6,14].

Recent advances in low-field ^1^H-NMR and micro-XRF spectroscopy have enabled spatially resolved analysis of crosslink density (CLD) and curative migration [14,15,16,17,18,19]. These methods have demonstrated that sulfur can penetrate deep into GTR particles and create localized differences in mechanical stiffness and network homogeneity [6,14]. Moreover, temperature scanning stress relaxation (TSSR) offers insight into the dynamic and temperature-dependent rearrangement of network structures, especially when recycled material phases participate chemically in the vulcanization process [5,14,20,21,22].

In light of these developments, previous work investigated a novel approach in which GTR is used as a direct substitute for the inactive filler calcium carbonate, keeping the total filler content constant [2]. This contrasts with prior research that typically adds GTR on top of existing formulations [2]. The focus lies on assessing whether GTR can serve not only as a space-filling component but also as a reactive, functional phase that contributes to the formation and modification of the elastomeric network.

This approach not only offers a new angle on circular rubber design but also provides a framework for evaluating performance-driven filler substitution strategies in industrially relevant EPDM sealing formulations. By redefining the role of GTR as a potentially interactive phase rather than inert waste-derived filler, the study aims to contribute to more sustainable and functionally optimized rubber compound development [2,4,8,11].

However, for the industrial application of such compounds, it is not sufficient to demonstrate reactivity and network integration alone. The long-term stability and aging behavior are decisive factors in evaluating whether this substitution strategy can succeed. Thermo-oxidative degradation of EPDM is known to change the material properties over time by increasing crosslink density, hardness, and tensile strength, along with a concomitant decrease in elongation at break, which indicates simultaneous crosslinking and chain scission processes. Aging also involves oxidative chemical changes, as demonstrated by an increase in carbonyl content, which reduces flexibility and thermal stability [23,24,25]. While EPDM is generally regarded as aging-resistant, GTR derived from end-of-life tires contains a heterogeneous mixture of aging-sensitive polymers like natural rubber (NR), butadiene rubber (BR), and styrene–butadiene rubber (SBR). The influence of GTR substitution on the aging behavior of EPDM compounds has not yet been systematically investigated, which is the focus of the present study.

## 2. Materials and Methods

### 2.1. Compounding

In this study, a model recipe (Table 1) of a sealing compound based on EPDM was used. The calcium carbonate, used as an inactive filler, was substituted in 25% increments with ground tire rubber K0002 (MRH Mülsen GmbH, Mülsen, Germany), referred to as GTR, made by cryogenic grinding of end-of-life tires. Previous studies have shown that the use of this GTR is advantageous because it allows better bonding to the matrix due to its small particle size, resulting in a lower reduction in mechanical properties than when using GTR with larger particle sizes [1]. The total amount of the inactive filler added up to 110 phr (parts per hundred rubber).

The compounds were processed in a laboratory scale internal mixer (GK 1.5 E, Werner & Pfleiderer, Stuttgart, Germany) in two stages, both starting at 60 °C and 50 rpm. In the first stage, all ingredients except sulfur, accelerators, and retarders were mixed for 10 min with intervals for ventilation. The second stage, conducted the next day, involved mixing the masterbatch for 1 min, then adding the crosslinking system and mixing for a total of 4 min. Subsequently, 2 mm thick test slabs were vulcanized at 180 °C for 8 min at 150 bar using a laboratory press (Drive series, 25 tons closing force, 250 × 250 mm chrome-plated steel press plates, Gibitre Instruments S.r.l, Bergamo, Italy).

### 2.2. Raw Material Characterization

The amount of free sulfur in the GTR was measured via conductivity changes before and after Soxhlet extraction with dichloromethane. Therefore, the specimens were burned with oxygen at 1350 °C to create sulfur dioxide, which was channeled into diluted sulfuric acid containing hydrogen peroxide. The change in conductivity was proportional to the sulfur content [1]. Additionally, the particle size distribution was measured using a laser diffraction particle size analyzer (Mastersizer 3000, Malvern Panalytical Ltd., Malvern, UK) and compared to that of the calcium carbonate. Both were also observed via digital optical (VHX 770, Keyence Corp., Osaka, Japan) and scanning electron microscopy (SU3900, Hitachi High-Tech Corp., Tokyo, Japan). SEM-EDX analyses on the GTR were performed using a scanning electron microscope (SU5000, Hitachi High-Tech Corp., Tokyo, Japan) equipped with an energy-dispersive X-ray spectroscopy (EDX) detector (Xmas80, Oxford Instruments, Abingdon, UK).

### 2.3. Compound Characterization

Curing tests were carried out with a Rubber Process Analyzer (RPA Flex, TA Instruments, New Castle, DE, USA) at 180 °C for 10 min to determine the vulcanization behavior of the compounds.

Tensile properties were measured on S2 dumbbell-shaped specimens in accordance with DIN 53504 [26], utilizing the 10 kN all-round table-top universal testing machine (Z010, Zwick Roell, Ulm, Germany) equipped with a 10 kN load cell. Tear resistance was determined on Graves angle test pieces following DIN ISO 34-1 [27] under identical testing conditions. Shore A hardness was measured on 6 mm thick stacked specimens, according to DIN ISO 48-4 [28], using a hardness tester (38009, Karl Frank GmbH, Weinheim, Germany).

The compression set was evaluated after 22 h at 100 °C in a laboratory oven (T6060, Heraeus Holding GmbH, Hanau, Germany), following DIN ISO 815 standards [29]. 

The swelling degree was measured by equilibrium swelling in cyclohexane (provided by Carl Roth). Samples were immersed until equilibrium swelling was reached. The swelling degree was determined gravimetrically according to Equation (1), where m_dry_ is the mass of the dry specimen and m_swollen_ is the mass after equilibrium swelling.

(1) $$Q(\%)=\frac{m_{swollen-}m_{dry}}{m_{dry}}\cdot 100$$

Anisothermal stress relaxation behavior was analyzed using a Temperature-Scanning-Stress-Relaxation-Meter (TSSR-Meter, Brabender GmbH, Duisburg, Germany). The samples for the TSSR measurements, approximately 2.2 millimeters thick, were heated from 23 °C to 300 °C at a heating rate of 0.033 K/min, with an isothermal relaxation time of two hours. The crosslink density was calculated from the rubber elasticity theory based on the stress-strain relationship in the rubber-elastic plateau region obtained during the TSSR measurements.

Thermal aging was performed in a laboratory oven (T6060, Heraeus) at 70 °C, 100 °C and 125 °C for different durations, as summarized in Table 2. The aging temperatures were chosen to cover the application cases for EPDM compounds, which is 100 °C as the upper service temperature, 70 °C as a temperature that is more probable because the compounds are seldom used on the limit, and 125 °C as higher temperatures should also be possible for a short time. For 125 °C, the durations were calculated under the premise that a 10 K temperature increase halves the reaction time, aligning them with the aging durations at 100 °C. Mechanical tests were also conducted on the aged materials. FTIR spectra were recorded using an ATR-FTIR spectrometer (Nicolet iS10, Thermo Fisher Scientific Inc., Waltham, MA, USA) equipped with a diamond crystal. Spectra were collected in the range of 4000–650 cm^−1^ with a resolution of 4 cm^−1^ by averaging 32 scans per measurement. One representative spectrum was recorded per sample. Baseline correction was applied using the instrument software (OMNIC, Version 8.0).

Additionally, low-field nuclear magnetic resonance (^1^H NMR) was performed on the reference and 100% GTR compounds in the unaged and most aged state (178.2 h at 125 °C) for measuring the crosslink density. NMR measurements were conducted using a Minispec mq20 spectrometer from Bruker Biospin GmbH (Billerica, MA, USA) at a constant temperature of 363 K (resonance frequency: 20 MHz).

## 3. Results

### 3.1. Raw Material Characterization

The amount of free sulfur is 0.02%, which can react to form new, additional crosslinks. However, it is known that sulfur accumulates around GTR particles, impoverishing the matrix and therefore reducing the crosslink density [6].

**Figure 1:**
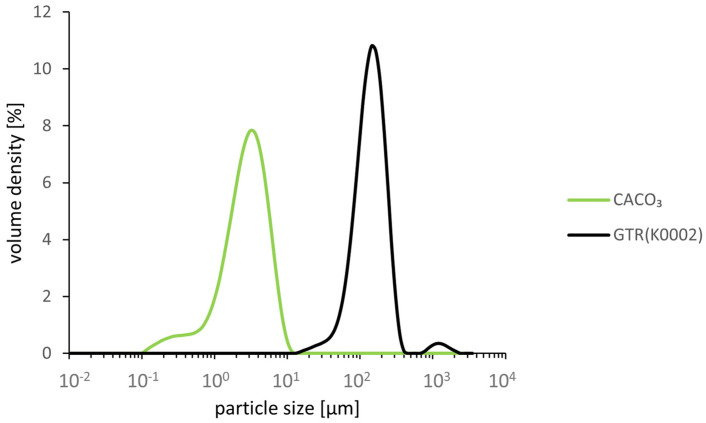
Particle size distribution (weight-based).

The GTR was sieved to achieve a mean particle size of 200 µm, which can be confirmed by the particle size measurements. A second, lower peak indicates particles with a size above 1000 µm; these are presumably agglomerated particles that could not be separated during preparation or elongated particles that passed through the mesh along their thinner dimension. The chalk particles are significantly smaller and have a mean particle size of 2.98 µm (see Figure 1).

EDX analysis of the GTR confirmed the expected composition. The corresponding spectrum is provided in the Appendix A.

**Figure 2:**
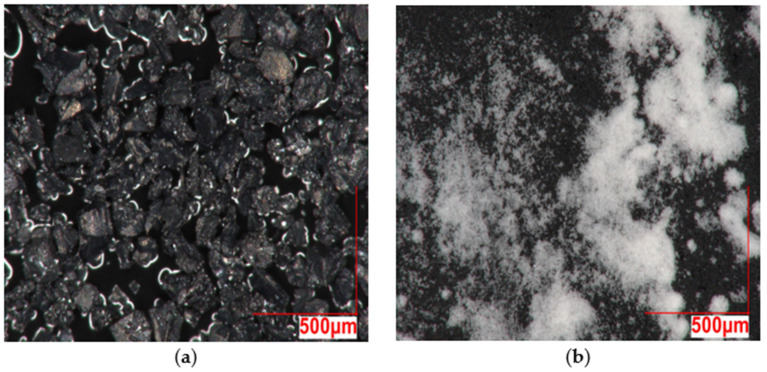
(**a**) Digital microscopy image at 0.2 k magnification showing GTR; (**b**) digital microscopy image at 0.2 k magnification showing CaCO_3_.

**Figure 3:**
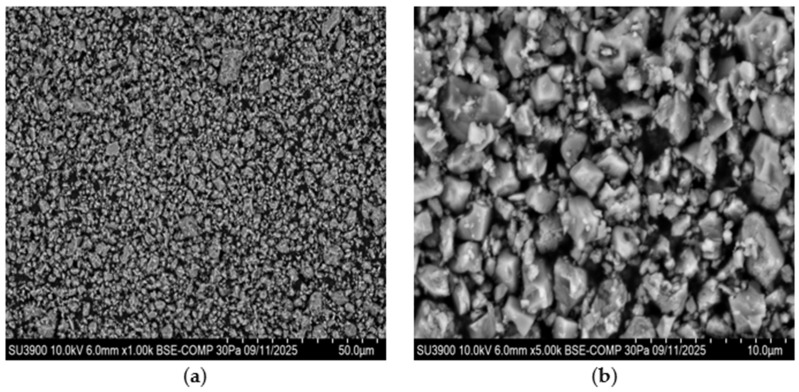
(**a**) Scanning electron micrograph of CaCO_3_ showing the morphology at 1.00 k magnification; (**b**) scanning electron micrograph of CaCO_3_ showing the morphology at 5.00 k magnification.

**Figure 4:**
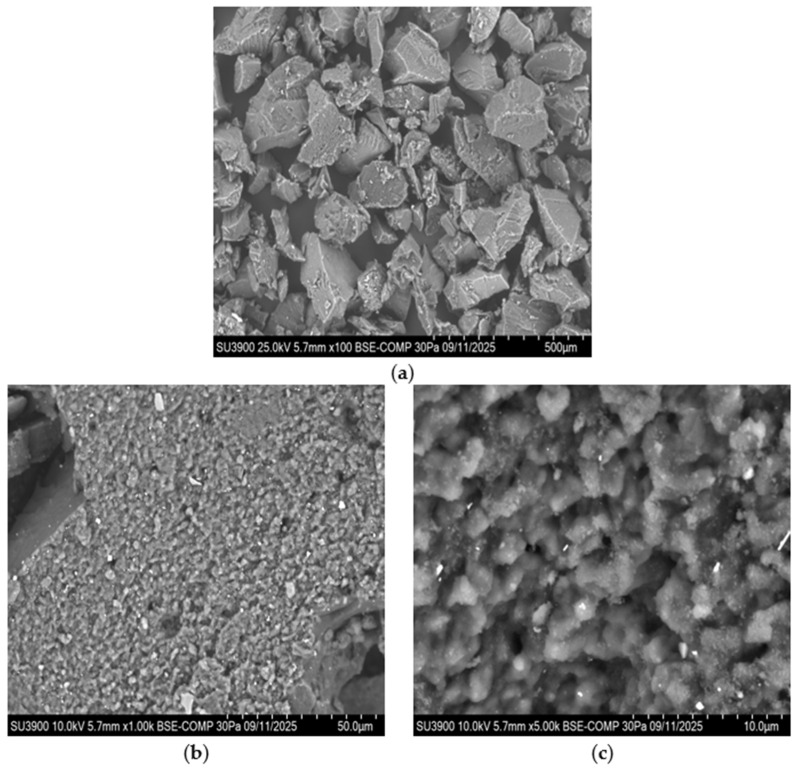
(**a**) Scanning electron micrograph of GTR showing the morphology at 0.10 k magnification; (**b**) scanning electron micrograph of GTR showing the morphology at 1.00 k magnification; (**c**) scanning electron micrograph of GTR showing the morphology at 5.00 k magnification.

The GTR exhibits irregular, rough, and porous surfaces, while calcium carbonate particles display faceted and angular morphologies (see Figure 2a,b). At 1.0 k magnification (see Figure 3a), a largely homogeneous particle distribution can be seen. The particles form a relatively uniform surface without distinct agglomerates. The CaCO_3_ particles exhibit irregular shapes with distinct edges. In some cases, smaller particles are attached to larger ones, indicating a limited agglomeration tendency (see Figure 3b). At 0.1 k magnification (see Figure 4a), the irregular particle size and shape of the GTR can be seen. At 1.0 k magnification (see Figure 4b), the sharp-edged particles become more clearly visible. The surface structure is porous and shows numerous cavities and microdefects. At 5.0 k magnification (see Figure 4c), the surface topography of the GTR particles becomes visible. The surfaces appear highly fissured with fine pores and irregular elevations.

### 3.2. Compound Characterization

#### 3.2.1. Curing

**Figure 5:**
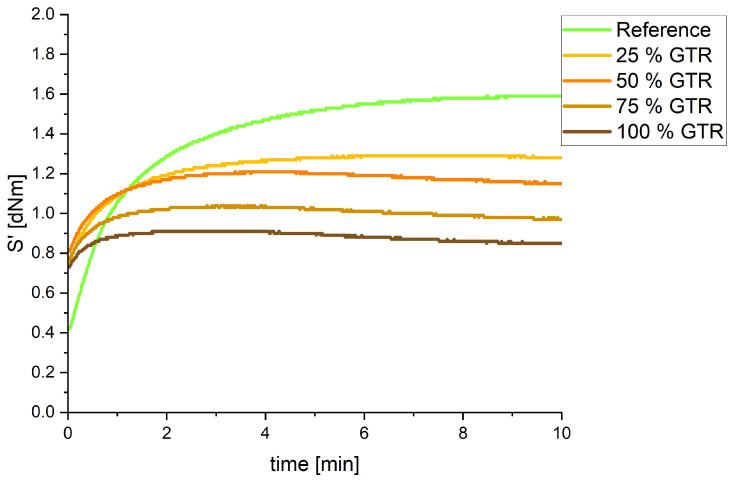
Curing curves of all compounds at 180 °C.

The curing tests show significant changes between the reference compound and the GTR compounds. While the reference compound has an initial torque around 0.4 dNm, the use of GTR increases it to nearly twice the value, to 0.7–0.8 dNm (see Figure 5), due to the presence of solid GTR particles, which contribute to the overall stiffness of the mixture. With an increasing amount of GTR, the change in torque ∆S decreases, which indicates reduced crosslink densities (see Table 3). For GTR amounts of 50% and more, there is even a visible regression of the curve (see Figure 5), which shows a destruction of the network. This behavior can be attributed to the migration of sulfur and curatives into the GTR, which reduces the availability for uniform network formation in the matrix. GTR also reduces crosslinking time t_90_ drastically from over 3 min of the reference down to only 0.3 min for 100% GTR. Unreacted crosslinking curatives from the GTR particles may migrate to the polymer matrix and accelerate the curing process [7].

#### 3.2.2. Crosslink Density

**Figure 6:**
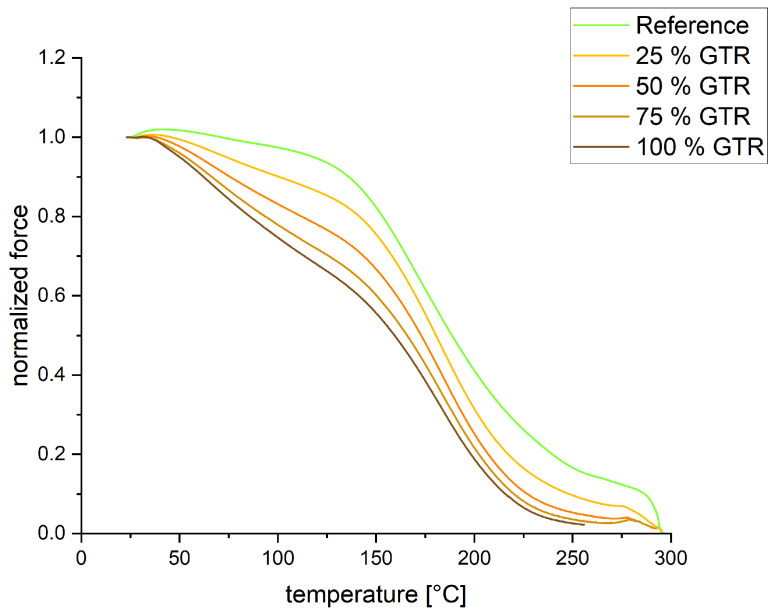
TSSR—normalized force curves.

**Figure 7:**
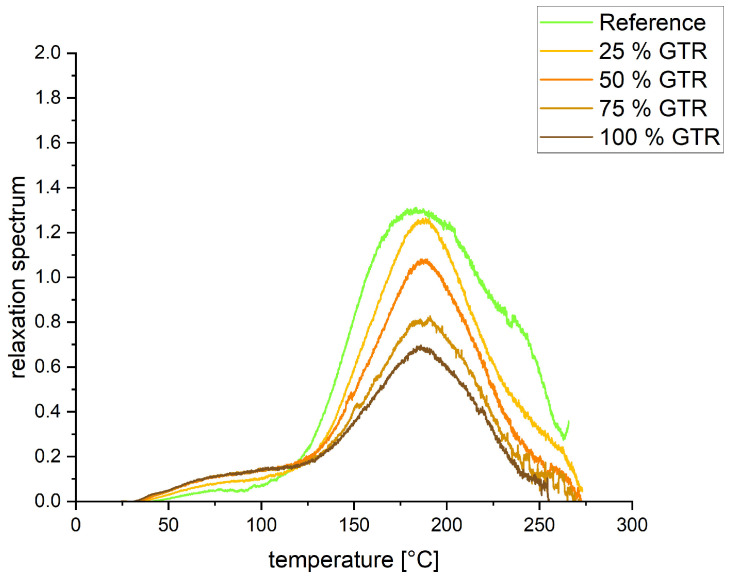
TSSR—relaxation spectrum curves.

The TSSR graphs show the normalized force (see Figure 6) and the relaxation spectrum (see Figure 7) over the temperature for all compounds. In Table 4, values from the TSSR measurements are summarized. With the TSSR meter, only the crosslink density of the reference could be calculated. Therefore, the normalized curve has to increase first before decreasing with higher temperature. The increase is attributed to the Gough–Joule effect, which describes the entropy elastic behavior of pre-stretched rubber when it is heated. In the beginning, the Gough–Joule effect is predominant over effects like thermal expansion or destruction, leading to a rise in the force. The GTR compounds show lower entropy elasticity than the reference. The initial stress σ_0_ decreases with increasing amounts of GTR; this value is correlated to hardness and tensile strength. T_50_, the temperature at which the normalized force is reduced by 50%, also decreases with increasing amounts of GTR, indicating poorer thermal stability. The relaxation spectrum exhibits even more insights into the materials. The reference shows an initial peak around 75 °C, where the physical separation of polymer from filler takes place. The GTR compounds are more likely to have a plateau from 75 °C to 125 °C, which may indicate poorer bonding of polymer chains to the GTR particles. Further, it can be seen that the peaks at around 180 °C decrease in height as the GTR amount increases. As the peaks of all compounds are nearly at the same temperature, the network structure is similar, while the height of the peaks indicates fewer crosslinks. This means that increasing the amount of GTR decreases the crosslink density, which is consistent with the findings of the curing measurements. The peak shoulder observed at approximately 240 °C for the reference sample suggests the presence of mono- and disulfidic bonds. A precise structural classification can only be made through additional verification, such as application of the thiol–amine method [30,31]. In the presence of GTR, this peak disappears, indicating a shift toward mainly polysulfidic bonds.

**Figure 8:**
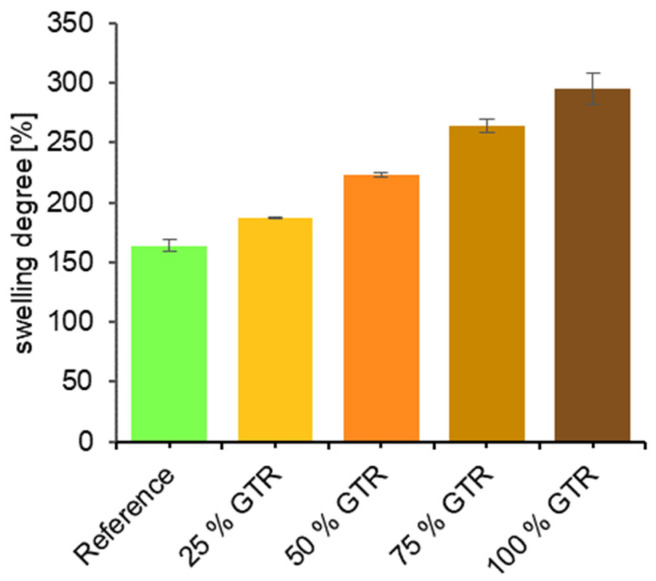
Equilibrium swelling—swelling degree.

Equilibrium swelling of the compounds shows that GTR decreases the crosslink density (see Figure 8). GTR was used as a substitute for the inactive filler calcium carbonate. Contrary to calcium carbonate, GTR swells in cyclohexane, which leads to more solvent migration into the samples. In addition, the use of GTR causes sulfur to migrate into the GTR particles, resulting in lower crosslinking of the matrix [6].

#### 3.2.3. Mechanical Properties

**Figure 9:**
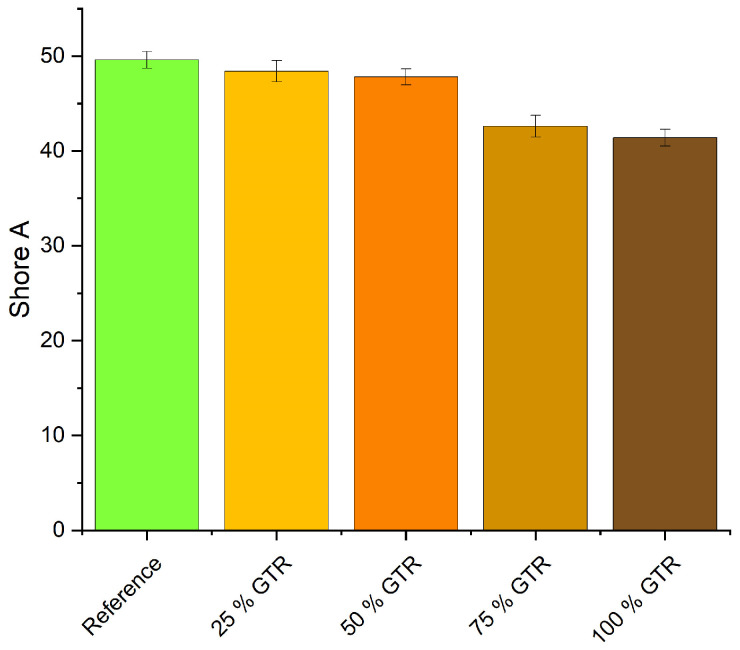
Shore A hardness.

**Figure 10:**
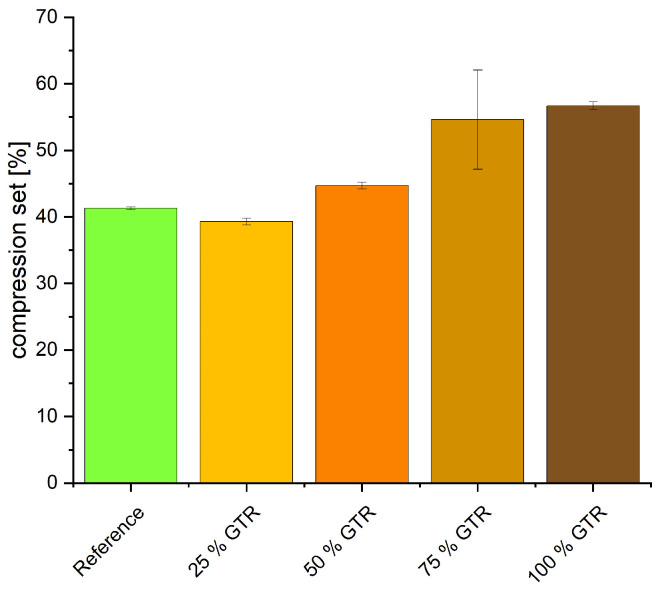
Compression set (22 h at 100 °C).

Shore A hardness decreases with increasing amounts of GTR (see Figure 9). This can be explained by the lower crosslink density and lower hardness of GTR particles compared to calcium carbonate. The compression set of the reference is 41%; the compound with 25% GTR yields a lower compression set of 39%, whereas with higher GTR content, the compression set rises to 57% (see Figure 10). As the compression set is measured after storage at elevated temperatures, there is a change in the network structures. Polysulfidic crosslinks convert into mono- and disulfidic crosslinks, effectively keeping the material in this new state. This leads to a higher compression set. These results are consistent with the TSSR findings showing the modification of the network towards more polysulfidic bonds.

**Figure 11:**
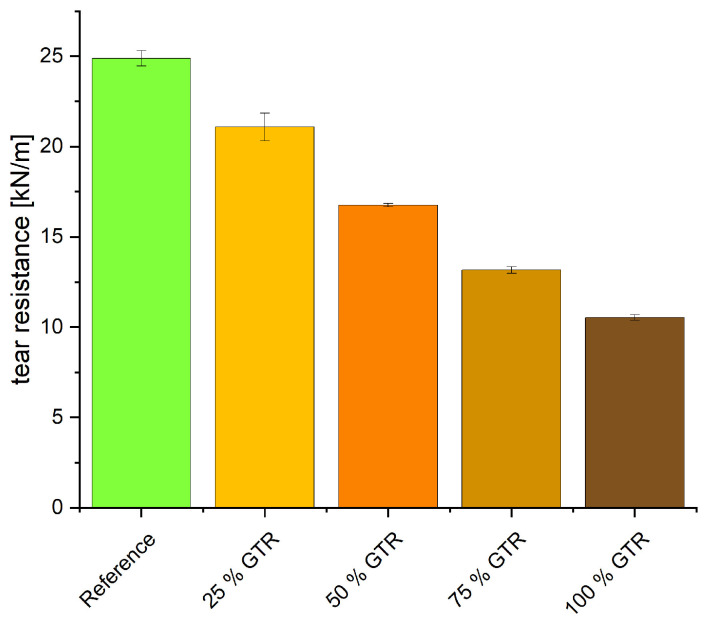
Tear resistance.

**Figure 12:**
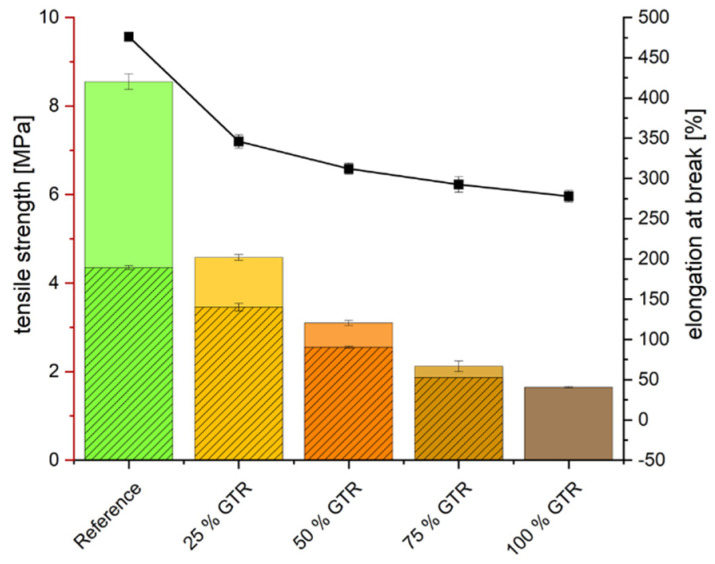
Tensile properties: tensile strength (colored bar), tensile stress at 300% elongation (striped bar), and elongation at break (line diagram).

GTR decreases the tear resistance significantly (see Figure 11). The particles of GTR act as defects in the matrix and impoverish the matrix of sulfur, which leads to an overall reduced material strength, which can also be seen in the tensile strength and elongation at break (see Figure 12). This decrease in tensile strength can also be partly attributed to the geometric effects resulting from the larger particle size of GTR compared to CaCO_3_. Large GTR particles act as stress concentrators and reduce the efficiency of stress transfer within the EPDM matrix, leading to premature failure [32]. In addition, the lower density of GTR increases the effective filler volume at constant phr, which further contributes to the deterioration of mechanical properties. A quantitative separation of particle size and volumetric effects would require dedicated experiments and is therefore beyond the scope of this study. The use of only 25% GTR nearly halves the maximum tensile strength from 8.6 to 4.6 MPa; with 100% GTR, only 20% of the original tensile strength remains.

The approximately additive property trends result from the gradual substitution of CaCO_3_ by heterogeneous GTR at constant phr. At low GTR contents, inactive domains are still limited, leading to continuous rather than abrupt changes in mechanical properties.

#### 3.2.4. Aging

**Figure 13:**
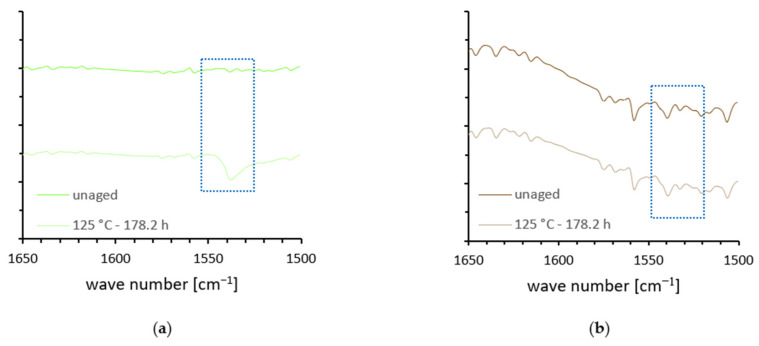
(**a**) Section from selected ATR-FTIR spectra of the reference compound at different aging stages; (**b**) section from selected ATR-FTIR spectra of the 100% GTR compound at different aging stages. The dashed blue boxes highlight the spectral region associated with the asymmetric stretching vibration of carboxylate groups (~1550 cm^−1^).

**Figure A1:**
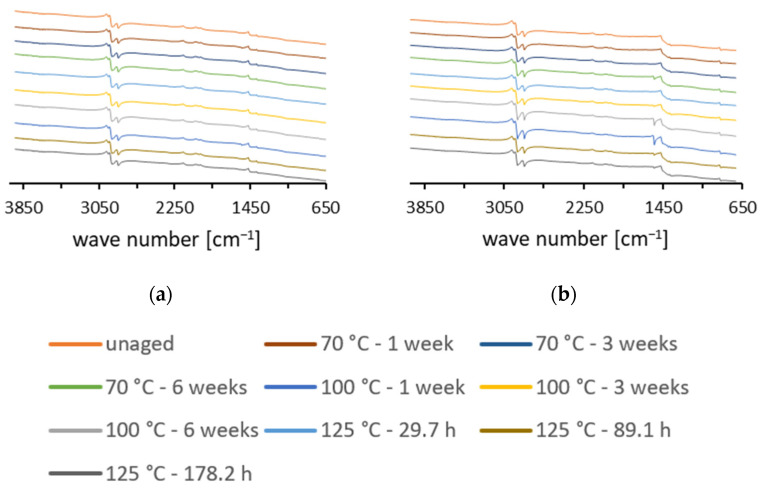
(**a**) ATR-FTIR spectra of the 100% GTR compound at all aging stages; (**b**) ATR-FTIR spectra of the reference compound at all aging stages.

To evaluate the aging characteristics of the compounds, the infrared spectra (FTIR) of both the reference compound and the 100 % GTR compound at various aging intervals are given in the Appendix (see Figure A1). In the reference compounds, differences between the spectra of the various aging states can be observed in the region around 1550 cm^−1^ (see Figure 13). This could be explained due to the formation of zinc carboxylates during the aging process. In rubber formulations, zinc oxide (ZnO) is commonly used as an activator, while stearic acid or other fatty acids serve as co-activators [33]. Thermo-oxidative aging of EPDM leads to the formation of oxygen-containing functionalities (e.g., hydroperoxides that can subsequently yield carboxylic acids) [34]. Free carboxylic acids may react with ZnO or Zn^2+^ ions to form zinc carboxylates [35]. Metal carboxylates typically exhibit an asymmetric COO^−^ stretching vibration at approximately 1550 cm^−1^ and a symmetric counterpart near 1400 cm^−1^ [36]. However, the detectability of these bands strongly depends on their concentration and on spectral superposition effects. In complex rubber formulations, overlapping absorption bands and baseline variations can obscure low carboxylate concentrations, which explains why the intensity of the carboxylate-related bands is not consistent across all aging states. These effects are inherent to complex polymer matrices and therefore apply equally to pure EPDM and to mixtures of EPDM and GTR.

In contrast to the reference compounds, the GTR-containing compounds show less pronounced spectral changes upon aging. This behavior is attributed to the fact that GTR originates from end-of-life tires and therefore already contains pre-aged domains. As a result, additional aging-induced chemical changes are less distinct in the FTIR spectra, as newly formed oxidation products do not significantly alter the overall spectral signature. While the analysis focuses on the 1650–1500 cm^−1^ region associated with carboxylate formation, aging mechanisms in GTR-containing compounds may also manifest in other spectral regions, such as carbonyl bands near 1720 cm^−1^ [37]. However, no significant aging-related changes were observed in these regions.

**Figure 14:**
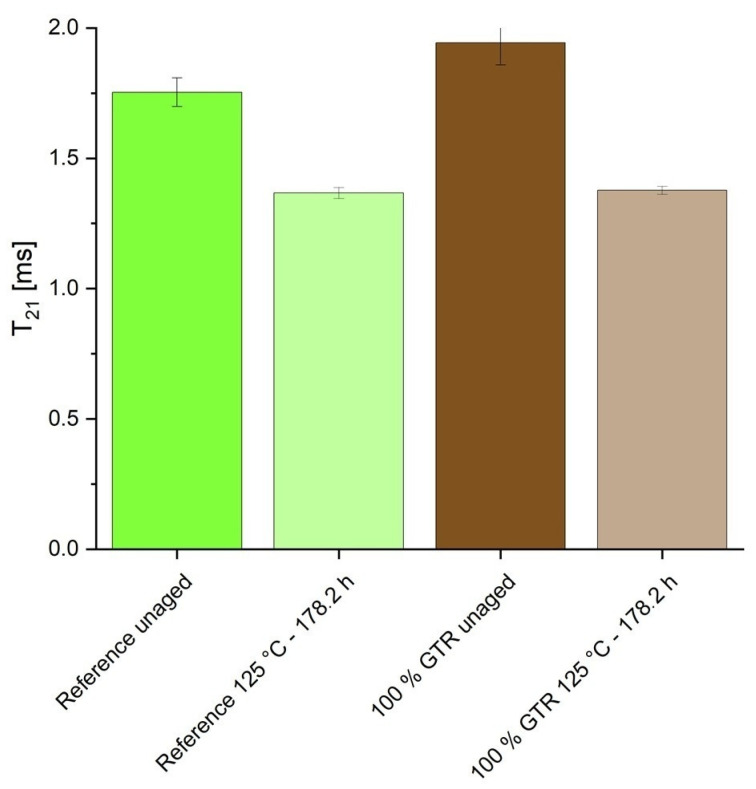
T_21_ relaxation time of the unaged and most aged samples.

The NMR results are given in Figure 14. The relaxation time T_21_ describes the proton mobility. A lower T_21_ value indicates lower mobility. Crosslinking influences the mobility; an increase in crosslinks leads to lower mobility. In the unaged state, GTR exhibits a higher T_21_ than the reference and therefore a lower crosslink density, which was already shown in the previous measurements. After aging at 125 °C for 178.2 h, the T_21_ is reduced, and both compounds have the same T_21_ value. The crosslink density increases due to aging; polysulfidic bonds change into mono- and disulfidic bonds.

**Figure A3:**
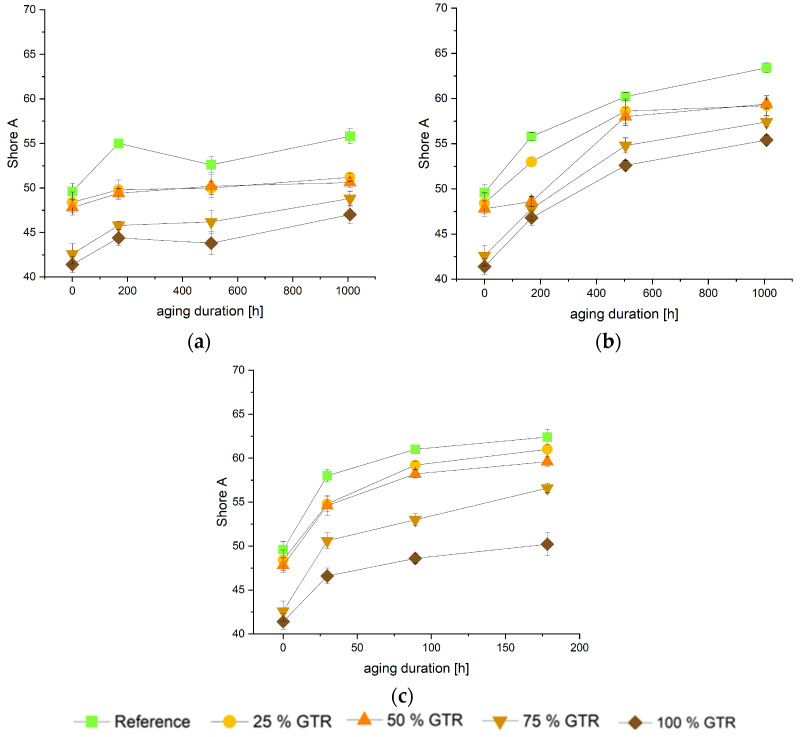
(**a**) Shore A hardness of the compounds after aging at 70 °C; (**b**) Shore A hardness of the compounds after aging at 100 °C; (**c**) Shore A hardness of the compounds after aging at 125 °C.

**Figure A4:**
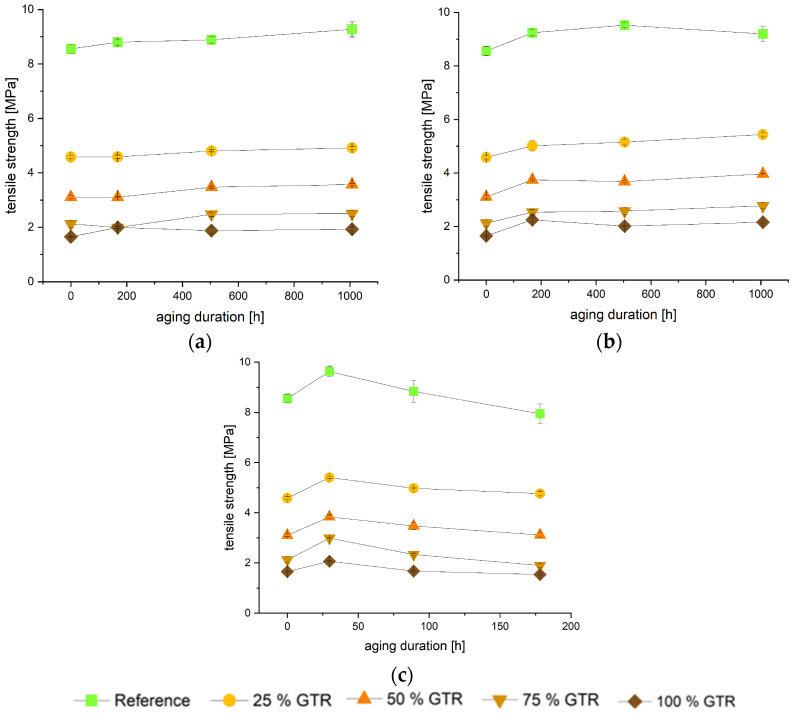
(**a**) Tensile strength of the compounds after aging at 70 °C; (**b**) Tensile strength of the compounds after aging at 100 °C; (**c**) Tensile strength of the compounds after aging at 125 °C.

**Figure A5:**
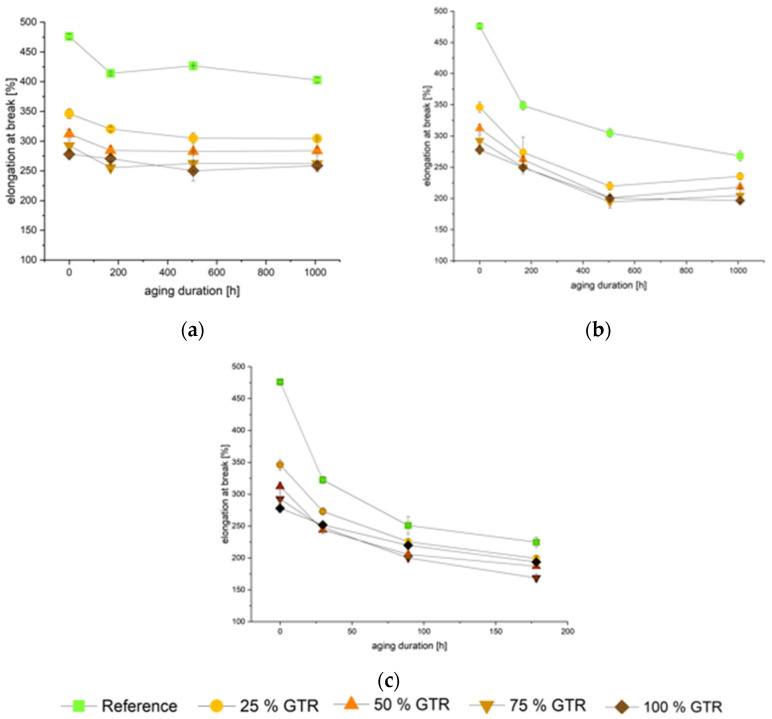
(a) Elongation at break of the compounds after aging at 70 °C; (**b**) Elongation at break of the compounds after aging at 100 °C; (**c**) Elongation at break of the compounds after aging at 125 °C.

**Figure A6:**
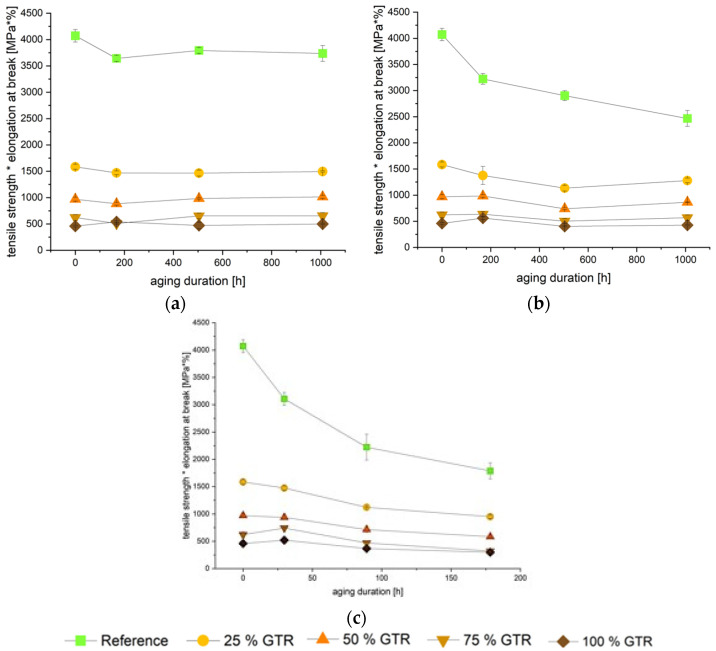
(**a**) Tensile strength × Elongation at break of the compounds after aging at 70 °C; (**b**) Tensile strength × Elongation at break of the compounds after aging at 100 °C; (**c**) Tensile strength × Elongation at break of the compounds after aging at 125 °C.

**Figure A7:**
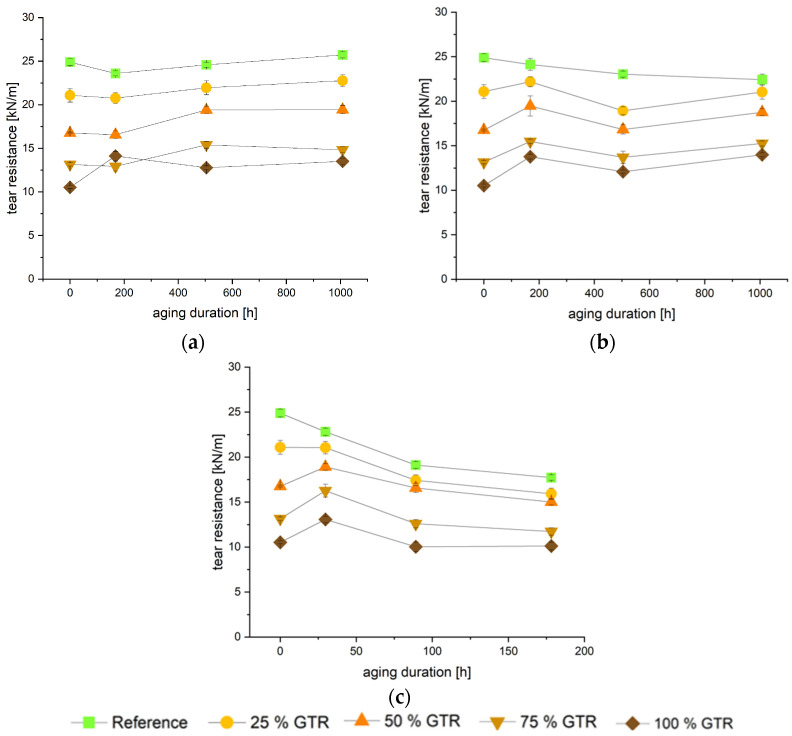
(**a**) Tear resistance of the compounds after aging at 70 °C; (**b**) Tear resistance of the compounds after aging at 100 °C; (**c**) Tear resistance of the compounds after aging at 125 °C.

**Figure A8:**
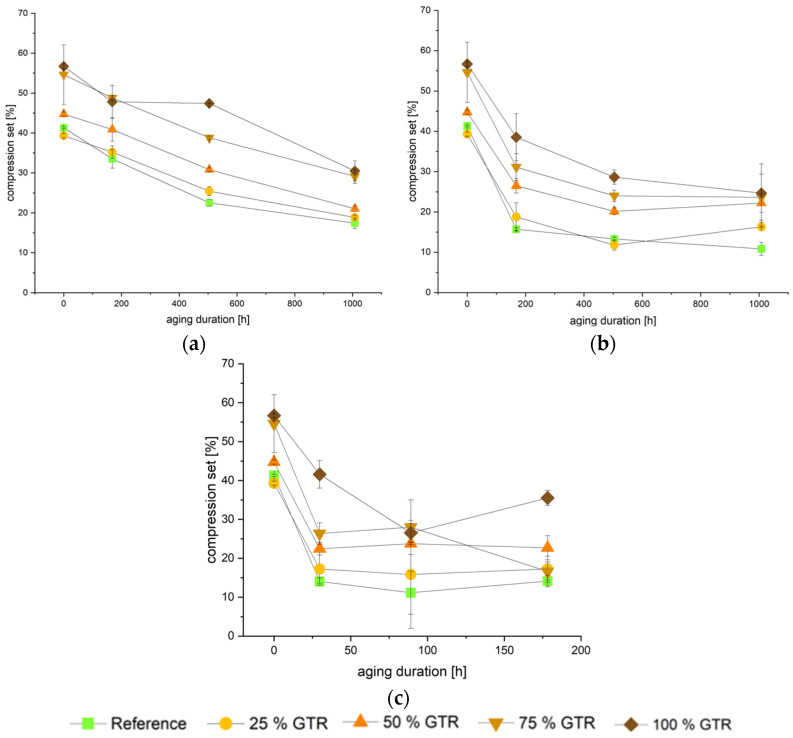
(**a**) Compression set of the compounds after aging at 70 °C; (**b**) Compression set of the compounds after aging at 100 °C; (**c**) Compression set of the compounds after aging at 125 °C.

The mechanical properties of both the reference compound and the 100 % GTR compound at various aging intervals are given in the Appendix A (see Figure A3, Figure A4, Figure A5, Figure A6, Figure A7 and Figure A8) to improve clarity and readability. With increasing temperature and aging time, the deterioration of the compound properties increases. Due to the change in crosslink structure over the aging period, the Shore A hardness increases for all compounds. The reference compound shows a change from 50 (unaged) to 63 (6 weeks at 100 °C), and the GTR compounds show similar changes from the unaged to higher aging states. The tensile strength increases for all compounds at 70 °C except the 100% GTR compound, which has a maximum in tensile strength after one week. At an aging temperature of 100 °C, the reference compound also starts to deteriorate after three weeks. Aging at 125 °C leads to a decrease after 29.7 h for all compounds. Overall, the tensile strength of the reference is always at least double that of the tensile strength of the GTR compounds due to the lower crosslink density, but the changes are similar to or even lower than in the reference compound without GTR.

The elongation at break decreases during aging at all temperatures and times. As the aging duration increases, the elongation at break of the reference sample approaches that of the samples containing GTR, thereby reducing the difference between these samples. The product of tensile strength and elongation at break show the same degradation of the tensile properties.

The tear resistance of the reference decreases with increasing aging time, with the exception of the test at 70 °C, where an increase in tear resistance is observed after 200 h. Similarly, the 100% GTR mixture at 70 °C shows behavior that deviates from the general trend, as the tear resistance after 200 h exceeds the value of the 75% GTR mixture.

The compression set decreases for all compounds similarly and reaches a plateau after aging at 125 °C, which can be explained by a change in the crosslinks during aging. An exception is the 100% GTR mixture, which has no plateau at a test temperature of 125 °C and exhibits an increasing compression set in the final aging state.

## 4. Discussion

The incorporation of ground tire rubber (GTR) leads to a reduction in crosslink density (CLD), which is mirrored in the resulting mechanical properties. This reduction is attributed to residual curatives and sulfur species present in the GTR, which may migrate from the EPDM matrix into the GTR particles, reducing the amount of sulfur available for crosslinking. Accelerated curing is attributed to accelerators inherently present in the GTR. As the proportion of GTR increases, Shore A hardness decreases, which correlates with the reduced CLD. Simultaneously, the compression set increases due to alterations in the network structure. Due to the reduced sulfur content in the polymer matrix, the sulfur-to-accelerator ratio shifts, favoring the formation of a higher proportion of polysulfidic crosslinks. During compression set testing, these polysulfidic crosslinks are less stable and may cleave, subsequently rearranging at other double bonds along the polymer chains. This cleavage and rearrangement process converts part of the network into shorter mono- and disulfidic crosslinks. The newly formed crosslinks tend to stabilize the specimen in its compressed state, thereby contributing to the increase in compression set [38].

A significant drop in tear resistance is observed, as GTR particles act as structural defects within the matrix, weakening the material’s mechanical integrity, particularly tensile strength and elongation at break. Therefore, the particle can be the initial point for crack formation. At a GTR content of 25%, only approximately half the original tensile strength is retained. This contradicts the findings of previous studies, in which a GTR content of 33% could be achieved without adjusting the recipe [2]. Although the same GTR particles were used in both studies, this difference in mechanical properties could have been caused by a different mixing process, which influences dispersion, crosslinking, and thermal damage. This can be verified by conducting reproduction runs with systematically varied mixing times and temperatures, followed by testing of the mechanical properties. NMR analyses support this trend, indicating a lower CLD in GTR-containing blends. Aging processes further increase the CLD due to continued transformation of polysulfidic crosslinks.

Parts of the deterioration in mechanical properties can be attributed to the particle size and distribution of the GTR used. A reduction in particle sizes is therefore preferable, as finer particles increase the specific interface area, improve phase dispersion, and, as a consequence, can promote the transfer of mechanical loads to the matrix [39].

An increase in surface roughness caused by gamma rays and the formation of polar functional groups can generally improve the adhesive properties of GTR, making this a good option for the studied approach [7]. In addition, devulcanization of GTR particles has proven effective, since the partial cleavage of sulfur crosslinks generates free chain ends and reactive sites. These can form new bonds during vulcanization, thereby enhancing compatibility and adhesion to the polymer matrix. However, it should be noted that uncontrolled or excessive devulcanization may lead to main-chain scission and deterioration of the intrinsic mechanical properties of GTR [40,41].

From a processing perspective, replacing CaCO_3_ with GTR influences compound processability due to coarser GTR particles. The larger particle size may facilitate initial incorporation and potentially improve extrusion behavior, while at the same time leading to an inferior surface finish compared to fine mineral fillers. Therefore, partial replacement of CaCO_3_ appears to be a more promising approach than complete substitution.

It has been demonstrated that GTR does not act as an inert filler but exhibits reactive behavior. GTR influences crosslinking through residual sulfur and accelerators, as well as sulfur diffusion into the particles. The relevance of these findings lies in the exploration of a novel approach to developing sustainable alternatives for elastomers, by replacing calcium carbonate (CaCO_3_), a conventional inactive filler, with GTR. 

The mechanical data are difficult to compare directly due to the formulation approach. Since the study was industry-oriented, GTR proportions were not adjusted for density differences relative to CaCO_3_. A more analytical approach, less practice-driven approach would involve correcting the GTR content with respect to its lower density. Reducing density enables weight reductions, which is particularly important in automotive and aircraft construction. Another main difference between the CaCO_3_ and the GTR is in the particle size, the GTR particles being much larger, which is also responsible for deteriorated mechanical properties.

## 5. Conclusions

The incorporation of GTR into EPDM compounds results in a measurable reduction in crosslink density, which is consistently reflected in the mechanical properties. Increasing GTR content leads to lower hardness, higher compression set and reduced tensile and tear strength.

The results demonstrate that GTR influences the vulcanization kinetics and the resulting network. Consequently, GTR-filled compounds differ fundamentally from calcium carbonate-filled reference materials in terms of crosslink structure.

The key finding of this study is that the aging behavior of GTR-containing compounds is comparable to, or in some cases even superior to, that of the reference compound.

From an application-oriented perspective, the partial substitution of calcium carbonate by GTR represents a viable approach to increase recycled content while maintaining acceptable mechanical performance and aging resistance. Higher GTR contents impose limitations related to particle size and processability, indicating that optimization of formulation and processing is required.

Additional investigations should include density-adjusted substitution strategies and the evaluation of lower GTR contents (e.g., 5%, 10%, 15%, and 20%).

The partial substitution of calcium carbonate by GTR provides a viable approach to increase recycled content and add value to end-of-life tires. This demonstrates the potential of GTR to contribute to more sustainable EPDM compounds, although further optimization is required to balance performance and processability.

## Figures and Tables

**Table 1:** Compound recipes.

	Reference	25% GTR	50% GTR	75% GTR	100% GTR
Unit			[phr]		
EPDM	100	100	100	100	100
N550	110	110	110	110	110
Calcium Carbonate	80	60	40	20	0
GTR	0	20	40	60	80
ZnO	3	3	3	3	3
PEG 4000	2	2	2	2	2
Stearic Acid	0.5	0.5	0.5	0.5	0.5
Softener	85	85	85	85	85
Calciumoxide	8	8	8	8	8
Sulfur	0.8	0.8	0.8	0.8	0.8
Accelerators	4.2	4.2	4.2	4.2	4.2
Retarder	0.1	0.1	0.1	0.1	0.1

**Table 2:** Applied aging states.

Temperature [°C]	Duration		
70	1 week	3 weeks	6 weeks
100	1 week	3 weeks	6 weeks
125	29.7 h	89.1 h	178.2 h

**Table 3:** Curing properties.

Sample	S’ max [dNm]	∆S’ [dNm]	t_90_ [min]
Reference	1.59	1.17	3.33
25% GTR	1.29	0.54	1.55
50% GTR	1.21	0.40	0.90
75% GTR	1.04	0.27	0.55
100% GTR	0.91	0.18	0.28

**Table 4:** TSSR properties of the different compounds.

Sample	Crosslink Density [mol/m^3^]	σ_0_ [MPa]	T_50_ [°C]
Reference	114	0.47	188.6
25% GTR	n. a.	0.38	180.5
50% GTR	n. a.	0.33	172.9
75% GTR	n. a.	0.28	165.8
100% GTR	n. a.	0.26	159.6

n. a. = not applicable.

**Figure A2:**
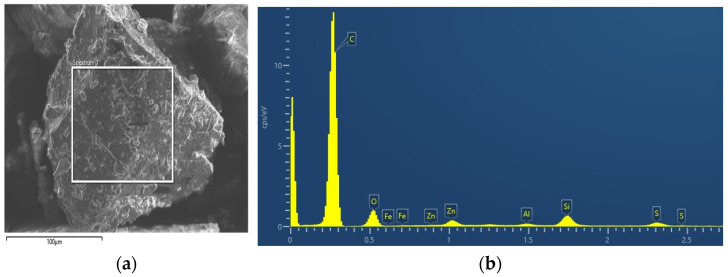
(**a**) Measuring area for the GTR tested; (**b**) EDX mapping of the GTR.

## Data Availability

The raw data supporting the conclusions of this article will be made available by the authors on request.

## Abbreviations

The following abbreviations are used in this manuscript:

GTR	Ground tire rubber
TSSR	Temperature scanning stress relaxation
CLD	Crosslink density
ELT	End-of-life tire
NMR	Nuclear magnetic resonance
FTIR	Fourier transform infrared spectroscopy
ATR	Attenuated total reflectance
n. a.	Not applicable
